# Advancing Immunotherapies for HPV-Related Cancers: Exploring Novel Vaccine Strategies and the Influence of Tumor Microenvironment

**DOI:** 10.3390/vaccines11081354

**Published:** 2023-08-11

**Authors:** Anna Jéssica Duarte Silva, Ingrid Andrêssa de Moura, Marco Antonio Turiah Machado da Gama, Lígia Rosa Sales Leal, Samara Sousa de Pinho, Benigno Cristofer Flores Espinoza, Daffany Luana dos Santos, Vanessa Emanuelle Pereira Santos, Matheus Gardini Amancio Marques De Sena, Maria Da Conceição Viana Invenção, Larissa Silva de Macêdo, Pedro Luiz de França Neto, Antonio Carlos de Freitas

**Affiliations:** Laboratory of Molecular Studies and Experimental Therapy—LEMTE, Department of Genetics, Federal University of Pernambuco, Recife 50670-901, Brazil; anna.jessica@ufpe.br (A.J.D.S.); ingrid.andressa@ufpe.br (I.A.d.M.); marco.turiah@ufpe.br (M.A.T.M.d.G.); ligia.leal@ufpe.br (L.R.S.L.); samara.pinho@ufpe.br (S.S.d.P.); benigno.cristofer@ufpe.br (B.C.F.E.); daffany.luana@ufpe.br (D.L.d.S.); vanessa.emanuelle@ufpe.br (V.E.P.S.); matheus.gardini@ufpe.br (M.G.A.M.D.S.); maria.conceicao@ufpe.br (M.D.C.V.I.); larissa.smacedo@ufpe.br (L.S.d.M.); pedro.francaneto@ufpe.br (P.L.d.F.N.)

**Keywords:** papillomavirus, TME, vaccines, cell therapy, TAM, APC

## Abstract

The understanding of the relationship between immunological responses and cancers, especially those related to HPV, has allowed for the study and development of therapeutic vaccines against these neoplasias. There is a growing number of studies about the composition and influence of the tumor microenvironment (TME) in the progression or establishment of the most varied types of cancer. Hence, it has been possible to structure immunotherapy approaches based on therapeutic vaccines that are even more specific and directed to components of TME and the immune response associated with tumors. Among these components are dendritic cells (DCs), which are the main professional antigen-presenting cells (APCs) already studied in therapy strategies for HPV-related cancers. On the other hand, tumor-associated macrophages are also potential targets since the profile present in tumor infiltrates, M1 or M2, influences the prognosis of some types of cancer. These two cell types can be targets for therapy or immunomodulation. In this context, our review aims to provide an overview of immunotherapy strategies for HPV-positive tumors, such as cervical and head and neck cancers, pointing to TME immune cells as promising targets for these approaches. This review also explores the potential of immunotherapy in cancer treatment, including checkpoint inhibitors, cytokine immunotherapies, immunotherapy vaccines, and cell therapies. Furthermore, it highlights the importance of understanding the TME and its effect on the design and achievement of immunotherapeutic methods.

## 1. Introduction

The Human Papillomavirus (HPV) is responsible for about 30% of cancers related to infectious agents (Bravo et al., 2010). It belongs to the Papillomaviridae family and infects epithelial cells of the skin and oral and genital mucosa. More than 280 types of HPV have been described, with approximately 200 capable of infecting humans, of which 12–16, 18, 31, 33, 35, 39, 45, 51, 52, 56, 58, and 59 are responsible for malignant neoplasias [1]. It is transmitted mainly sexually, and its infection is associated with most cervical cancers and other carcinomas, such as anogenital, head and neck, conjunctival squamous cell carcinoma, and genital warts in men and women [2,3].

Through the years, observing this HPV/cancer relationship, preventive measures against the virus were developed. Currently, five prophylactic HPV vaccines are licensed for use, ranging from bivalent, tetravalent, and nonavalent, protecting at least against the main oncogenic HPV types 16 and 18 [4]. However, there is still resistance to prophylactic vaccination against HPV concerning the stigmatization of sexual infection, cultural beliefs, and lack of universal health coverage in some countries [5]. In addition, the available vaccines do not induce protection against all persistent high-risk HPV types, which are capable of causing precancerous lesions, and the risks involved with conventional treatments for invasive lesions such as ablation and excision became a challenge for developing countries [1]. Thus, it is critical to develop therapeutic vaccines to benefit infected people that possess lesions caused by HPV [6].

Knowledge of the cells and metabolites involved in the post-HPV and tumor microenvironment allows its immunomodulation as a therapeutic strategy and the development of drugs for this purpose [7,8]. In addition, a better understanding of the relationship between immunity and cancers, especially those related to HPV, has allowed the study and development of therapeutic vaccines against these neoplasias [9]. These vaccines use different technologies to deliver an immunogenic load of the virus, mainly related to E6 and E7 proteins, to antigen-presenting cells to induce a cytotoxic immune response against HPV-infected cells [10]. Some vaccine approaches are based on viral peptides, live vectors, nucleic acids, and whole cells, in addition to therapeutic strategies for HPV-related cancers such as T-cell transfer and T-cell immune checkpoint inhibitors [11,12]. Here, we discuss the characteristics of HPV-related cancers and present well-established mechanisms of immunotherapies and new strategies under study to control these cancers within the perspective of the influence of the tumor microenvironment on the design and success of these approaches.

## 2. Cancers Related to HPV Infection and Classic Treatment Strategies

HPV may be classified primarily in relation to its oncogenic potential, being divided into high and low risk. This classification is largely related to the association of each type with the development of lesions and/or malignant or benign tumors [13,14]. Infections caused by low-oncogenic risk HPVs are usually self-limited, ranging from asymptomatic infections to the appearance of warts and benign tumors known as papillomas [15]. However, high-oncogenic risk HPV infections have the potential to trigger the development of cancer, especially when associated with persistent infection and predisposing factors [16,17].

Cervical cancer is strongly associated with HPV infection, as this is a preponderant condition for its development [18], being the third cancer that most affects women in the world [19]. However, other cancers of the anogenital tract such as the vulva, vagina, anus, rectum, and penis may have their development associated with HPV infection [17,20,21,22,23]. It is estimated that 25% of head and neck cancers are positive for HPV infection [24,25], a percentage that may be even higher at close to 42% [26], and include sites such as the nasal and oral cavities, tonsils, pharynx, and larynx [27,28,29]. Although with less evidence available, HPV infections have also been observed in middle ear cancers [30,31,32] and, because of their location and association with low-risk HPV, deserve attention regarding their participation in neoplastic development.

The detection of HPV DNA has been repeatedly reported in lung tumors worldwide [33,34,35,36], and while the association between infection and tumor progression is unclear, a recent study developed by Wang and collaborators investigated the possible interference of oncoproteins E5, E6, and E7 of HPV-16 transfected in H292 cells in the expression of the nuclear epidermal growth factor receptor (EGFR), where higher levels of EGFR were associated with greater sensitivity to cisplatin, a chemotherapeutic widely used to treat cancers [37].

There is an ongoing debate about the association between HPV infection and breast cancer development. Although there is no evidence of a direct relationship between the presence of HPV and neoplastic developments in breast tissues, some studies indicate that HPV may aid in the initiation of tumor development with a subsequent absence of viral detection related to elimination by the immune system [38,39]. Furthermore, the expression of oncoproteins E6 and E7 during HPV infection in breast tumors has already been correlated with a decrease in BRCA1 and BRCA2 expression, possibly aiding in tumor progression [40]. It is worth noting, however, that a 2016 study by Lawson and colleagues reported HPV presence in malignant breast tumors in women previously treated for cervical cancer, with both sites positive for the same type of HPV, suggesting that transmission may occur through circulatory and lymphatic systems [38].

Classic treatments for these cancers vary according to the affected site and stage of tumor development. In general, HPV-related cancers are treated like those not related through classic treatments such as chemotherapy, radiotherapy, and excision of tumor tissue [41,42]. However, HPV-positive head and neck cancers may be preferentially treated by radiation, due to their higher radiosensitivity [43,44,45].

Lesions classified as precancerous in the vagina, vulva, anus, and penis can be treated using laser therapy, cryosurgery, and surgical removal [41,46,47,48]. As for advanced neoplastic lesions of the uterus, cervix, and cervical canal, electrosurgical loop excision (LEEP) or cold conization techniques are used [49,50]. For both techniques, the removal of the abnormal tissue and part of the adjacent tissue is performed in a cone-shaped cut, a procedure known as conization [51]. In cases of persistent neoplastic lesions of the cervix and uterus for which previous treatments have not led to total tumor elimination, total hysterectomy is performed as a treatment [52].

With advances in understanding the intricate factors involved in tumor genesis, growth, and immune evasion, the development of immunotherapies as prospective alternatives to traditional cancer treatments has become feasible [53,54,55]. It is no different, therefore, that such knowledge also applies to HPV-related cancer. These studies make use of antigens specific to HPV, such as the E6 and E7 oncogenes [56,57,58,59], the immune checkpoints molecules such as the protein programmed cell death protein 1 (PD-1) and its ligand programmed death ligand 1 (PD-L1) [60], to whole cell therapies [61,62]. However, further studies are needed to understand and design immunotherapies against HPV-related cancers.

## 3. Immunotherapy and Cancer

Immunotherapy is a biological therapy that aims to restore or enhance the ability of the immune system to prevent and fight disease [63]. In recent decades, it has become a powerful clinical strategy for the treatment of some types of cancer, such as melanoma, kidney cancer, and cervical cancer, among others [64,65,66]. This therapy has shown potential in the complete and lasting regression of tumors, even in advanced or metastatic stages of the disease, by increasing the activity of immune cells, such as T cells and natural killer (NK) cells, which are responsible for identifying and destroying abnormal cells [67].

Cancer cells adapt cellular mechanisms, thereby avoiding immune system checkpoints and facilitating the development of the tumor microenvironment [68]. Checkpoint inhibitors are used as methods of immunotherapy, using monoclonal antibodies directed at CTLA4 and PD-1, proteins receptors that are located on the cell membrane of T cells and cancer cells. Such inhibitors work by blocking signals from cancer cells, allowing the immune system to attack and destroy these cells [69,70]. This targeted approach has proven effective in treating various types of solid tumors and malignant hematologic tumors [64,70]. Cytokine immunotherapies are another promising approach for cancer treatment. These proteins aid in the regulation and direction of the immune system. They are synthesized in a laboratory and injected into patients at higher doses than those produced naturally [71]. Cytokines slow or even stop tumor growth by increasing the immune system’s response to cancer. Although cytokine therapy has significant side effects, it has been effective in treating various types of cancer [71].

Therapeutic vaccines can be used as an approach that aims to stimulate the immune system to recognize and attack cancer cells [72]. These vaccines, which can be made from the patient’s tumor cells or specific proteins found in cancer cells, have shown promising results in certain types of cancer, such as prostate cancer and cervical cancer [73,74]. Cell therapies such as chimeric antigen receptor (CAR)-T and CAR-NK are the latest advances in this field, offering a new paradigm for cancer treatment. Chimeric antigen receptor (CAR) therapy has shown efficacy in clinical trials in the treatment of Merkel cell carcinoma (MCC), a rare and aggressive form of skin cancer [75]. Clinical trials using human lymphocytes genetically engineered to express Merkel cell polyomavirus-encoded T antigens have demonstrated regression of established tumors following therapy [76]. However, T-cell therapies may have limitations due to the activation of tumor immune escape strategies and cytokine release syndrome in CAR-T cell therapy. However, studies using activated autologous or allogeneic NK cells seem to overcome these limitations, promoting the regression of these tumors in clinical trials for metastatic MCC [77]. MCC and cervical cancer share similarities because they are mainly caused by viral infections and affect epithelial tissues such as skin and mucous membranes. They are known to be immunologically responsive diseases, so the immune system plays a significant role against cancer cells. Therefore, this immunotherapy suggests advantages to treating these pathologies, releasing antitumor immunity against an immunogenic tumor, and may target cancers with similar characteristics.

Immunotherapy has revolutionized cancer treatment. Overall, these therapies modulate the activity of immune cells, such as T cells, through the adoptive administration of modified cells or monoclonal antibodies [78,79]. The number of approved immunotherapeutic drugs is growing, with methodologies in clinical and preclinical development [80]. These treatments are in stark contrast to conventional oncology therapies as they are more specific and have fewer side effects [69,74]. However, their large-scale implementation has been slow due to the need for controlled modulation of the immune system, as these therapies can have adverse effects, including autoimmunity and nonspecific inflammation [69]. Furthermore, for some types of cancers, only a fraction of patients benefit, highlighting the importance of comprehending the complexities of tumor biology, the tumor microenvironment, and the responsiveness of each agent to improve the current approaches or develop new strategies [81].

## 4. Study of the Tumor Microenvironment (TME) for the Development of Immunotherapies

The tumor microenvironment (TME) includes the presence of immune cells, stromal cells, blood vessels, and the extracellular matrix (ECM). These elements help in the survival, local tissue invasion, and metastatic spread of cancer cells [82,83].

Changes in the composition and concentration of ECM components such as collagen, proteoglycans, hyaluronic acid, and laminins are essential points, as they influence the prognosis of some types of cancer and can lead to resistance to systemic therapy [84]. In addition, the metabolism of cancer cells regulates the presence of immune cells and tumor progression. Thus, the degradation of glucose and glutamine by cancer cells acidifies the TME, which stimulates metalloproteinases and cathepsins to degrade the matrix, promoting the development of the invasive tumor phenotype [85]. In a murine model study, it was observed that the depletion of glucose alters the metabolism of T lymphocytes, which can prevent the production of effector molecules such as IFNγ, IL-2, and IL-17, and granzyme B, inhibiting the formation of antitumor T lymphocytes [86].

It was observed that there is a high presence of M2 macrophages, CD5hi cDC2 type dendritic cells, and low-density neutrophils (LDN) in TME, which not only contribute to cancer progression and metastasis but also stimulate the formation of Th2 immunosuppressive lymphocytes and Th17 [87]. In addition, tumor-associated fibroblasts (CAF) produce high levels of TGFβ that act by attracting regulatory T cells and polarizing macrophages to M2. Together, these cells negatively regulate the infiltration and activity of cytotoxic CD8+ T lymphocytes [84,88]. In addition, the presence of immunosuppressive factors such as VEGF, IL-10, TGF-β, and PGE2 in TEM inhibit DC maturation, generating tolerogenic DC that promote the development of Th2-type responses [87].

Finally, the plasticity of immune cells contributes to the formation process of new vessels in the tumor environment (angiogenesis), the production of factors such as VEGFA, EGF, FGF2, IL-8, CXCL12, TNFα, IL-1β, VEGF, TGFα, HGF, ANG1, CXCL1, CXCL8, CXCL9, CXCL10, CCL3, IL-10, Arg1, and galectin-1, stimulate this event [89]. Understanding and studying the tumor microenvironment has allowed for the development of new therapeutic strategies against cancer, such as immune system stimulators (IL-2 and INF-α), therapeutic vaccines, adoptive cell therapy, use of antibodies (anti-CTLA-4, anti-PD1, and anti-PD-L1) that trigger anticancer T responses, use of oncolytic virus therapies, and the combined use of these strategies as antagonistic models for cancer development [90,91,92,93]. 

## 5. Cytokines and Immune Cells Found in the TME of HPV-Related Cancers

Chronic HPV infection is associated with the induction of epithelial microenvironment remodeling and acts as a cofactor in infection persistence and disease progression [94]. The immune system cells of the tumor microenvironment include inflammatory cells such as macrophages, mast cells, neutrophils, lymphocytes, and NK cells [95]. The levels, molecular characterization, and ratio of these TME cells have been used as efficient prognostic biomarkers to indicate the degree of risk and treatment responsiveness of HPV-positive patients [96,97]. Although abundant in tumor tissue, mast cells do not affect the immune microenvironment or the growth of tumors induced by HPV [95]. In this sense, in this topic, we will address the action of macrophages, lymphocytes, neutrophils, and killer cells surrounding HPV-positive tumors.

### 5.1. Cytokines

Cytokines are crucial immunological mediators in the communication between immune cells, in addition to helping the immune response to infectious diseases and cancer [98], highlighting the interleukins 2 and 12 (IL-2, IL-12), interferons (IFNs), and TNF-α [99]. IL-12, secreted by dendritic cells and activated macrophages, is considered a promising target in the immunotherapy of HPV-related cancers because of its antitumor activity potentiation [99]. IFNs, as IFN-γ, have activity in creating an antiviral state from cell-mediated immunity enhanced by the presence of TNF-α [99,100].

The infection caused by HPV induces keratinocytes to secrete pro-inflammatory cytokines [94]. In persistent HPV infection, regulatory and pro-inflammatory cytokines are present, helping to associate local immune mediators with virus clearance [101]. In HPV-positive patients with head and neck cancers, levels of pro-inflammatory cytokines are higher than in HPV-negative patients [98].

Th1 cytokines are activators of the cell-mediated immune response and may allow for the elimination of HPV. On the other hand, Th2 cytokines impair the immune response and lead to the permanence of the virus and, consequently, chronic infection [99,102]. The change from Th1 to Th2 cytokine pattern was observed in patients with HPV-positive laryngopharyngeal cancer, associated with low levels of IL-2 and IFN-γ in advanced stages of the disease and increased levels of IL-2 and IL-12 in early stages [103]. In the same study, metastases were correlated with higher levels of IL-8 and IL-10 and a significant reduction of IFN-γ [103]. 

Pro- and antitumoral activities of TME-associated immunomodulatory cytokines can regulate the malignancy of head and neck tumors [104]. In HPV-positive patients with head and neck cancers, high expression of high mobility group protein B1 (HMGB1) and anti-inflammatory cytokines may indicate immune evasion and disease recurrence and be employed as prognostic biomarkers [98]. The cytokines TGF-β, IL-4, IL-6, and IL-10 contribute to the progression of infection and cancer development and are modulated by HPV oncogenes to create a Th2 microenvironment [99,105,106]. IL-10 is the most studied Th2 cytokine in the infectious and carcinogenic process due to the microenvironment favorable to tumor development, acting together with TGF-β [99]. 

Coexpression of cytokines including INF-γ and interleukin IL-17A, GM-CSF and monocyte chemoattractant protein-1 (MCP-1), GM-CSF and RANTES, IL-17A and RANTES, and MCP-1 and eotaxin has been associated with more severe cervical neoplasia in HPV-positive patients [107]. IFN-γ, GM-CSF, RANTES, and eotaxin expression increase significantly with disease worsening [107]. E6 and E7 oncoproteins of high oncogenic risk HPV types 16 and 18 inhibit the expression of IFN type I, also altering its activity in signal transduction pathways by blocking genes involved in immune surveillance and cytotoxic response [99,108]. IFN-γ is a known marker of cellular responses against tumors and HPV infection [109,110].

E6 and oncoproteins interact with the interferon regulatory transcription factor (IRF), inhibiting the transcriptional activity of cells, and also inhibiting the activation of TNF receptor-associated factor 3 (TRAF-3) by upregulating ubiquitin C-terminal hydrolase L1 (UCHL1) [94,111]. IL-6 is also a target of oncogenes E6 and E7 that upregulate its expression in keratinocytes and stimulate a chronic inflammation status in the tumor microenvironment [94,112].

Polymorphisms in cytokine genes that encode proteins involved in Th1 and Th2 cellular responses can be used as predictive biomarkers in the development of cervical cancer in HPV-positive patients [113]. The relationship between Th1 and Th2 cytokines can help to assess the prognosis of HPV-positive cancer patients [103].

### 5.2. Lymphocytes

Cervical tumors are infiltrated with CD4+ and CD8+ effector T cells, which can be suppressed or downregulated by regulatory T cells (Tregs). Tregs isolated from the tumor infiltrate are specific for E6 and E7 of HPV16 in cervical cancer and inhibit the secretion of cytokines such as IFN-γ and IL-2 produced by antitumor T cells [114]. Despite the high infiltration of CD8+ T cells in cervical lesions, they may not be sufficient to suppress malignant cell proliferation. High-risk HPV affects CD8+ T and memory CD8+ T cell activity, both in cell density and distribution [115,116]. An imbalance in Th1 and Th2 responses (Th2 > Th1), mediated by CD4+ helper T lymphocytes, may contribute to the immune dysregulation associated with infection promoted by high-risk HPVs [8]. 

T cells located in central and marginal regions of the tumor have antitumor potential. However, as the tumor progresses, the neoplastic cells become able to escape the immune activity of CD4+ T cells by modifying their surface antigens [117]. The Th17 cell (CD4+, IL-17+), a T cell phenotype involved in the inflammatory response, has been reported for its action in HPV-positive cancers [99,118]. CD8+ T cells are critical to the immune response in HPV-positive and HPV-negative tumors. These cells can recognize antigens presented by tumor cells and eliminate them directly, releasing pro-inflammatory cytokines and cytolytic granules.

Although the role of B lymphocytes in tumor immunity is less explored than T cells, they can promote tumor immunity mainly through IL-10 secretion [119]. B cells and plasma cells in the TME can also inhibit antitumor immunity through immunomodulatory proteins such as TGF-β, IL-10, and IL-35 [120]. Differences in tumor-infiltrating immune cells were observed in head and neck squamous cell carcinomas (HNSCC), with a higher frequency of intratumoral B cells present in HPV+ HNSCC and a higher frequency of dysfunctional CD8+ T cells in HPV-HNSCC, while CD4+ Treg suppresses the antitumor immune responses in TME in both HPV+ and HPV. Studies in lung cancer [121] and breast cancer [122] have suggested that B cells present in the TME correlate positively with overall survival in patients with HPV-positive tumors [123]. A population of B cells characterized by the overexpression of PD-L1, CD39, and Ly6A/E and by the negative regulation of molecules CD86, CD74, CD79a, and MHC II, independent of IL-10, was identified, suggesting new mechanisms of action in the process of tumorigenesis [119].

### 5.3. Neutrophils

Neutrophils are one of the immune cells that react more quickly to changes in the tumor microenvironment (TME) [124]. These cells are the final effectors of acute inflammatory responses, acting to eliminate extracellular pathogens [125]. Tumor-associated neutrophils (TANs) play relevant roles in modulating antitumor immunity [126]. Recently, it was observed that high levels of TANs and their associated secreted factors are essential for tumor immunosuppression, progression, and metastasis [127].

Studies have suggested that neutrophil infiltration and HPV status may be effective prognostic parameters for head and neck cancers [124,126,127]. A cohort study found higher levels of neutrophils in HPV-positive patients with oropharyngeal cancer, with lower survival rates and a higher risk of disease recurrence for these patients [128]. 

The neutrophil–lymphocyte ratio (NLR) indicates the balance between systemic inflammation and immunity, and its percentage in the blood has been suggested as a prognostic factor for head and neck cancers [97,129]. Elevated NLR values indicate more aggravating situations in patients with oropharyngeal squamous cell carcinoma HPV positive, and the evaluation of NLR before the treatment of head and neck cancers can allow the stratification of patients according to the risk group [97,130].

Neutrophils can act in tumor formation by releasing factors such as reactive oxygen species (ROS) and inhibiting effector function and proliferation of T cells [126,131]. The immunosuppressive phenotype, exhibited by neutrophils after the tumor’s microenvironmental signals, is observed in biopsies of patients with HNSCC and inhibits tumoricidal functions of natural killer cells by secreting transforming growth factor beta (TGF-β), nitric oxide, and arginase-1 [132]. A study in a murine model demonstrated that neutrophils may be helpful in tumor elimination and metastasis reduction from the combined actions of TNF-α, CD40 agonist, and tumor-binding antibody. Thus, neutrophils may be potent antitumor immune mediators from an inflammatory pathway that can be assessed in neutrophil-mediated cancer clearance [133]. In addition to exploring the role of neutrophils as an inflammatory and chemotactic mediator in the innate immune response, recent studies have pointed to roles related to antigen presentation, essential both for antitumor activity and for generating memory responses from interaction with CD4+ T cells [134,135,136].

Thus, neutrophils may play a dual role in tumor development and progression. However, further studies and evaluation of the potential application of cell therapy tools are needed to verify the use of neutrophils beyond a prognostic marker in related HPV tumors [126].

### 5.4. Natural Killer Cells

HPV-infected cells can be eliminated by NK cells that induce an inflammatory immune response, recruiting more NK cells, macrophages, dendritic cells, and NK T cells (NKT) to the site of infection. NK cells also mediate the activation of adaptive immune cells, such as CD4+ and CD8+ T cells [137]. Persistent infection with HPVs forces tumor-surrounding NK cells to downregulate their membrane receptors, such as NKp30, NKp44, NKp46, and NKG2D, leading to an impairment of the antitumor activities of NK cells. Furthermore, HPV16 oncoproteins E6 and E7 inhibit IL-18-dependent IFN-γ production in NK cells by suppressing IL-18 binding to its α-chain receptor [8].

Despite these mechanisms, NK cells produce large amounts of interferon gamma (IFN-γ), which plays a relevant role in activating the innate immune system and in the differentiation of helper T cells [138]. By influencing adaptive immunity, as well as their inherent tumor cytolytic ability, NK cells are the main effectors of antitumor immunity [139].

### 5.5. Macrophages

The tumor microenvironment harbors two main subpopulations of tumor-associated macrophages (TAMs): M1-like (which acts effectively to eliminate tumor cells) and M2-like (which stimulates tumor growth and progression). Thus, M1-like TAMs are related to prolonged survival, while M2-like TAMs indicate lower survival rates [96,140]. Macrophages are versatile cells derived from hematopoietic progenitors or monocytic cells, essential to activating local immunity [140]. A comparison between radiosensitive and radioresistant cells indicates an M1 macrophage polarization induced by IL-6 secretion in radiosensitive HPV-positive squamous cell carcinoma cells, indicating a significant presence of type 1 macrophages in radiation-sensitive cells [141].

Studies report that secretion of human heparin-binding epidermal growth factor (HB-EGF) by M2 macrophages enables radioresistance in head and neck squamous cell carcinoma [142]. These macrophages are involved in mechanisms of immunosuppression through the stimulation of Tregs and the secretion of cytokines such as TGF-β and IL-10, establishing a TME favorable for tumor progression [143]. The diverse functions of macrophages in response to HPV infection associated with tumor formation raise substantial interest in TAMs in immunotherapy approaches [140].

In cervical lesions, there is an increase in the infiltration of TAMs from CD163+ and CD68+ cells with lower IFN-y production and a restriction of the ability to promote T-cell proliferation [71]. Additionally, a greater expression of the cytokines IL-10, IL-17, IL-23, and TGF-β has been associated with high-risk HPV [144]. Additionally, the progression of precancerous cervical lesions is related to increased levels of IL-8, IL-β, IL-10, and IL-21 secreted by TAMs [145].

## 6. Immunotherapies Targeting HPV-Related Cancers

The commercially available prophylactic vaccines against HPV are composed of a recombinant version of the L1 viral capsid protein, expressed in the last stages of the viral cycle. These vaccines are based on neutralizing antibodies and immunological memory promoted by virus-like particles (VLPs). However, patients with HPV-related cancer do not produce viral particles because of the incipient L1 expression after HPV genome integration. Thus, L1-based vaccination does not have proven efficiency in infected individuals, especially in advanced stages [146,147,148,149]. In this scenario, it is necessary to invest in immunotherapy approaches aimed at HPV-related cancers, capable of inducing cellular immune responses relevant to control the carcinogenesis associated with the HPV infection.

**Figure 1:**
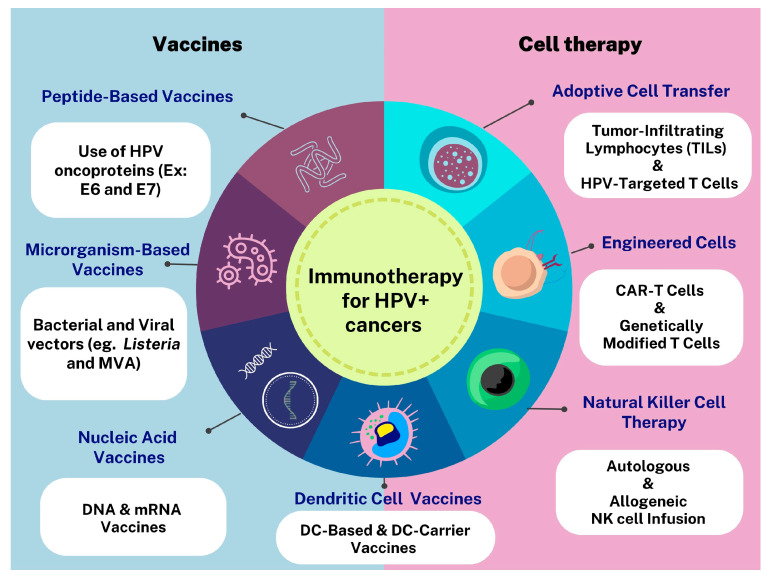
Immunotherapy for HPV+ cancers. This figure illustrates the various types of cell therapy and vaccine approaches used in the treatment of HPV+ cancers, including head and neck, cervical, and oropharyngeal cancers.

In general, the therapeutic vaccines against HPV, regardless of the stage of cancer development (initial or in metastasis), are based on E6 and E7 oncoproteins applied in different strategies, including subunit and nucleic acids vaccines, delivered by bacterial and viral vectors, and dendritic cell vaccines (Figure 1). In addition, other forms of immunotherapies are also in development, such as adoptive cell transfer (ACTs) and immune checkpoint inhibitors [12,149,150,151].

Peptide vaccines against HPV use sequences derived from E6 and E7 oncoproteins, with trials that have reached clinical phase II, evaluating the effects from the early stages of carcinogenesis to the development of metastasis in HPV+ tumors. One of the main limitations of these vaccines is their low immunogenicity, compared to other strategies, and the consequent need for adjuvants to increase the targeted immune response [12]. 

Immunotherapy based on the transfer of adoptive cells, in turn, showed promising results by demonstrating complete regression in patients with metastatic cervical cancer after infusion of reactive cytotoxic T lymphocytes to respond to E6 and E7 of HPV16 [110]. Another example is the phase I study carried out by PAPA et al. (2018), who used CAR-T cells against tumor cells of head and neck cancers without notification of toxicity [152].

Regarding immune checkpoint inhibitors, studies are focused on the production of antibodies that neutralize cell receptors of T lymphocytes, such as PD-1, PDL-1, and CTLA-4, for example. The expression of these receptors is upregulated in tumor cells to inhibit cell death that could occur naturally [153,154,155,156], favoring tumor development. Studies in this line are being developed by Moskovitz et al. (2018) with the combined use of chemotherapy with anti-CTLA-4, ipilimumab, for advanced cases of cervical cancer (NCT01711515) and cases of recurrence or metastasis (NCT01693783). The use of monoclonal antibodies is an interesting alternative, not only as immune checkpoint inhibitors but also for blocking receptors that, when activated, favor the progression of neoplasia, such as EGFR [157]. Table 1 summarizes the current state of the art regarding antibody-based strategies targeting EGFR, NKG2D, and other relevant targets for HPV-related cancers. It is noteworthy that certain strategies mentioned in the table are currently undergoing clinical evaluation for various types of cancer, including HPV-related malignancies.

## 7. Cell Therapy for HPV+ Cancers

Conventional treatment modalities for HPV+ cancers often face limitations, leading to exploring new therapeutic approaches [177]. Cell therapy, which involves using immune cells to target cancer cells, has emerged as a promising alternative to traditional cancer treatments [74]. Although incipient, several types of cell therapy have been investigated for HPV+ cancers, including dendritic cell therapy [178,179,180,181,182], NKs [10,183,184], and T cells [61,185,186,187] (Figure 1). Therefore, this topic aims to provide an overview of cell-based therapies for HPV-associated malignancies and discuss their implications for future therapeutic interventions.

Cell therapy involves the transfer of cells with the potential to repair or replace damaged tissue by using immune cells to target and destroy cancer cells [188]. In using T cells against HPV+ cancers, the therapies employed are classified into two main strands: modified immune cells and adoptive cell transfer [189]. Engineered immune cells can be obtained through gene editing technologies such as CRISPR-Cas9 to enhance their antitumor activity against HPV+ cancer cells or be genetically engineered to express CARs that recognize specific HPV antigens [10,190]. Although few HPV+ studies have used CRISPR-modified T cells, targeting HPV oncogenic genes has become more common in recent years with the increasing use of this technology [191]. For example, Chen et al. (2020) promoted, via cleavage of E6/E7 mRNAs by the CRISPR/Cas13a system, the inhibition of in vitro and in vivo growth of human cervical cancer cells [192]. Another approach involved using CRISPR-Cas9-carrying liposomes to enhance autophagy and immune activation related to cell death, promoting infiltration of HPV-targeted CD8+ T cells [193].

On the other hand, T cells transduced to express the E7-targeted TCR recognized and killed cervical and oropharyngeal cancer HPV16+ cell lines and mediated regression of established tumors in a murine model [185]. Additionally, the first phase I/II study using T cell receptor gene therapy induced regression of HPV-associated metastatic epithelial cancers [61]. CAR T-cell therapy has shown promising results in preclinical models and early-stage clinical trials for HPV+ cancers [187]. In a study by Zheng et al., DC-enhanced and SOCS1-silenced CAR-T-PD1 cells demonstrated potent antitumor activity in vitro and in vivo against cervical cancer cells [194]. In addition, two clinical trials use T cells expressing TCR HPV-16 E7 targeted at treating recurrent/refractory or metastatic-positive cancers (NCT05686226) and targeted at HPV-associated cancers (NCT02858310). As for ACT, studies use tumor-infiltrating lymphocytes (TILs) and T cells directed to HPV. TILs have shown promising results in treating advanced HPV+ cervical cancers [195,196]. In a phase II study, the adoptive transfer of TILs mediated the regression of lesions in HPV-associated epithelial cancers [186].

In addition to these applications, cell therapy using NK cells and dendritic cell vaccines has emerged as a promising strategy for treating HPV+ cancers. Cell-based therapies using NK cells and DC vaccines offer a promising avenue for targeted immunotherapy against HPV+ cancers [180,183]. NK cells exert potent cytotoxicity against tumor cells, while DC vaccines enhance antigen presentation and immune activation, and will be further explored in Session 8 of this review. A preclinical study by Veluchamy et al. investigated the use of allogeneic NK cells derived from umbilical cord progenitors, highlighting their potential to target and kill HPV+ cervical cancer cells [184]. In this sense, NK cells can recognize and eliminate transformed cells, exerting direct cytotoxicity [197,198]. 

These findings highlight the potential of cell therapy to target HPV-related malignancies, offering a viable option for cell-based treatment. However, there are still hurdles before cell therapy can be widely used to treat HPV+ cancers. For the successful application of CAR T and TIL cell therapy, specialized infrastructure, experience, and resources are required. Only a few specialist cancer facilities now provide CAR T cell therapy, and the expense of treatment is prohibitive for many patients [199,200]. This medical treatment can be hampered by cytokine release syndrome, which can cause symptoms such as fever and exhaustion, which are considered adverse effects [201,202]. In the scenario of HPV+ malignancies, the expression of antigens is heterogeneous, involving the expression of viral antigens and tumor-specific antigens [203].

Furthermore, they use a variety of methods to evade immune surveillance, including the down-regulation of antigen presentation machinery and the presence of immunosuppressive substances in the tumor microenvironment [204,205]. These processes can impede the detection and eradication of tumor cells, limiting the effectiveness of TIL and NK treatment. Combining this therapy with immunomodulatory drugs or immune checkpoint inhibitors, on the other hand, may help to overcome immune escape mechanisms [206]. Understanding the relationships between immune cells, the tumor microenvironment, and HPV+ malignancies can help identify potential targets and develop personalized therapy methods [143,207].

## 8. Antigen-Presenting Cells as Vaccine Targets for the Treatment of HPV-Related Cancers

Investigating the molecular and cellular tumor landscape has been fundamental to establishing effective and specific immunotherapies [208]. In this review, we have already addressed the cells in the microenvironment of tumors caused by HPV infection and how they can be markers for prognostic evaluation or even components of immunotherapy strategies. The development of therapies focused on favoring T cell-mediated responses is on the rise, especially those based on CAR-T technology [187]. On the other hand, antigen-presenting cells are also critical components of the antitumor immune response and have been studied not only for their expression profile in tumors but also as targets for immunomodulation, targeting of vaccine antigens, and even as whole-cell vaccines. For this reason, we will focus here on strategies targeting macrophages and dendritic cells in HPV-related tumors.

### 8.1. Modulation of Tumor-Associated Macrophages as an Immunotherapy Strategy

As already described, macrophages are one of the main TME immune cells in the most diverse types of cancer, including HPV-positive cancers. Their prognostic role has been pointed out for HPV-positive head and neck squamous cell carcinomas, even though further studies are needed to characterize this role and the appropriate markers for its characterization [96]. So far, studies with macrophages associated with HPV+ tumors are more focused on assessing their phenotype and prognostic value. Because functional data and assays targeting these cells as an immunotherapy target in approaches for HPV-positive tumors are scarce, this is a field of study to be explored. It is worth noting that most of the approaches presented here are still experimental and show promise for HPV-related tumors but have not yet been validated in vivo studies.

Monocytic myeloid-derived suppressor cells (mMDSCs) are immunosuppressive cells found in the TME and resemble macrophages classified as M2 [140]. These cells are recruited in the early stages of carcinogenesis mediated by infection caused by HPV, and their presence is associated with a worse prognosis for the evolution of the neoplasia, as well as resistance to response to chemoradiation [209]. Viral replication and the expression of oncoproteins E5, E6, and E7 contribute to tumor progression, immune response evasion mechanisms, and establishing an immunosuppressive environment [210]. Due to the functions performed in this microenvironment, mMDSCs or M2 macrophages are considered important as prognostic markers and targets of immunotherapy strategies of polarization to reach an antitumor response pattern [211].

Different molecules can promote this modulation, from cytokines such as TNF-α and IFN-γ to agonists for receptors such as TLRs 7 and 8, which act by activating Th1-type responses. This approach has been evaluated for HPV-positive HNSCC treatment [212]. The delivery of these agonists is generally done through nanoparticles and nanoemulsions to guarantee the receptor molecule interaction on the target cells [213,214]. Another interesting target is colony-stimulating factor 1 (CSF1), which plays a critical role in the new TAMs recruitment and the regulation of monocytic myeloid-derived suppressor cells based on binding to its receptor (CSF1R) [215]. Blocking the CSF1/CSF1R signaling pathway influences the response mediated by macrophages and T cells in carcinomas of mammary and cervical origin [216]. Thus, CSF1 may also be a possible target for modulation of its expression or receptor-binding in the context of TME.

One of the difficulties in establishing efficient immunomodulation approaches may be ensuring specific delivery to target TAMs. Therefore, it is essential to seek immunotherapy delivery vectors that interact with specific receptors and promote macrophage activation, in addition to the internalization of vaccine antigens that can be processed and presented to T lymphocytes [217]. These antigens may be, for example, oncogenes derived from HPV, which help generate specific antitumor responses.

**Figure 2:**
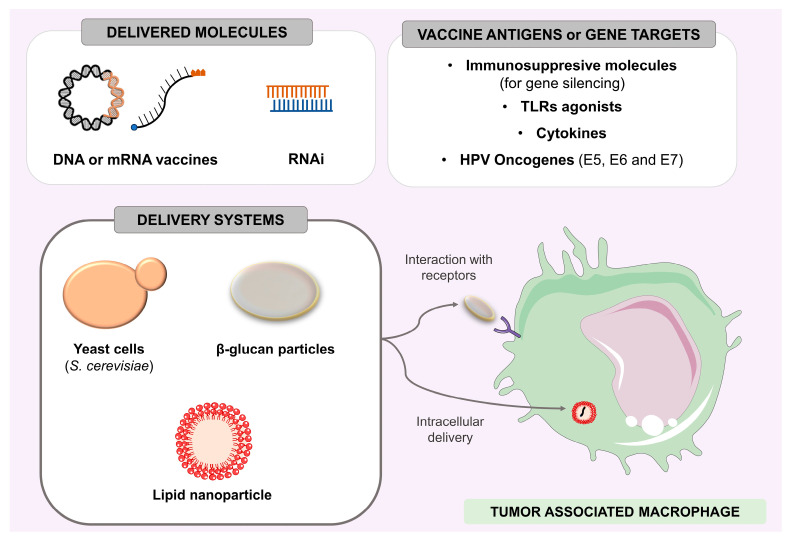
TAMs can be targets of gene therapy based on DNA or mRNA vaccines or even delivery of interfering RNA molecules. Several therapeutic genes can be used to improve the antigenic presentation of tumor antigens, polarize macrophages towards an antitumor profile (by silencing suppressor molecules or increasing the expression of Th1 cytokines), and increase the expression of receptor ligands that favor TME immunomodulation. Whole yeasts or β-glucans derived from their wall can polarize M2 macrophages are ideally sized to be phagocytosed by APCs, and act as vehicles for antigens in these therapeutic strategies. Similarly, nanostructured particles are also commonly used vehicles for antigenic delivery.

Yeasts and β-glucan particles can be suitable carriers for this approach due to the interaction of these vaccine vectors with specific macrophage receptors such as CD206 [218]. β-glucans have inherent immunostimulatory properties capable of polarizing macrophages to an M1 profile [219]. These particles have been evaluated as vehicles for DNA and RNA vaccines, exhibiting specific delivery to antigen-presenting cells and triggering effective immune responses [220,221,222]. Even yeasts such as *Saccharomyces cerevisiae* or particles derived from its wall components have shown promise for RNAi delivery [223]. An interesting approach with this delivery system is the construction of plasmids that allow the simultaneous delivery of DNA and RNAi vaccines [224]. Thus, it would be possible to develop strategies that employ the delivery of HPV oncogenes as antigens in DNA vaccines associated with RNAi sequences targeting markers such as CSF1, aiming at blocking neoplasias in their earliest stages. In addition, strategies based on E6 and E7 oncogenes in association with sequences that modulate the expression of cytokines, such as IL-10 and IL-6, could be designed once they are related to the induction of cell proliferation [225]. These cytokines are promising targets because their expression is often positively associated with the expression of viral oncogenes, thus acting together to promote and maintain dysregulated cell proliferation. Such approaches can both increase the immunogenicity of tumors and lead to an increase in antitumor activity [226]. The TAMs present in HPV-related carcinomas can be targets in gene therapy strategies (Figure 2), with a broad spectrum of intervention possibilities, such as (I) favoring the antigenic presentation of high-risk HPV oncoprotein epitopes to T lymphocytes; (II) delivery of cytokine genes capable of polarizing M2 to M1 macrophages; (III) inhibition of secretion of suppressor molecules; (IV) delivery of receptor agonists that signal Th1 immune response pathways; and (V) silencing of expression of genes encoding recruiter proteins of mMDSCs. Although these approaches have not yet been tested for HPV-positive tumors, they are alternatives that can complement immunotherapy strategies aimed at macrophages present in cervical cancer or head and neck carcinomas.

In addition to modulating the recruitment of immunosuppressive macrophages, immunotherapies can target suppressive molecules released by TAMs. Among the major suppressive molecules are TGF-β, IL-10, IL-6, GM-CSF, CCL2, CCL18, Arg1, PGE2, and IDO [140,227]. In general, strategies have employed RNAi to silence the expression of these mediators [228,229]. Acting by decreasing the local expression of these molecules is an indirect way of favoring the functions of macrophages and dendritic cells to activate cytotoxic CD8+ T responses and direct CD4+ T helper responses towards a pro-inflammatory profile [140].

In summary, therapy strategies focusing on the macrophages present in HPV-positive tumors may involve a reduction in the recruitment of TAMs, the polarization of M2 to M1 cells, the stimulation of antitumoral cytokine release (ex. TNF-α) [220], and the inhibition of immunosuppressive molecules secreted by TAMs [225,230]. These mechanisms can be developed from different platforms, from macrophages with chimeric receptors (CAR-M) to vaccines based on M2 macrophage elements as therapeutic targets [231,232]. However, until the publication of this review, these procedures are still little explored in the context of tumors related to HPV infection.

### 8.2. Dendritic Cell Vaccines

Dendritic cells play a crucial role in immunotherapy strategies due to their ability to mediate and induce both innate and adaptive immune responses [233]. They are designed to enhance antigen presentation through the activation and expansion of tumor-specific T cells inciting the induction of long-lasting antitumor immunity [180,234]. In addition, some subtypes of DCs acquire exogenous antigens and present them through MHC-II molecules for CD4+ T cells and MHC-I molecules for CD8+ T cells resulting in cross-presentation [235]. Different types of DC-based vaccines have been explored, and both ex vivo and in vivo models have shown the potential in stimulating specific immune responses. Specifically, they are considered promising targets for developing cancer vaccines, especially for HPV-positive tumors. 

In terms of vaccine administration, two main models have been used: ex vivo and in vivo. In the ex vivo model, DCs are isolated and cultured from monocytes or hematopoietic progenitors, stimulated to mature, and then injected into the patient’s bloodstream [236,237,238,239]. In contrast, in the in vivo model, DCs are stimulated within the body itself through various means, leading to recognition, internalization, maturation, and antigen presentation to T cells [240]. However, to fully comprehend the functionality and immune response elicited by DCs, it is also essential to consider the different cell subtypes. These subsets each play specific roles in the immune response, and their impact varies in the treatment of HPV+ cancers. The three main categories of DCs are conventional DCs (cDCs), monocyte-derived DCs (MoDCs), and plasmacytoid DCs (pDCs). The characteristics and unique impacts of these DC subtypes can be conveniently visualized in Table 2.

The application of DCs in cancer immunotherapy is mainly associated with their ability to capture and present tumor-associated antigens (TAAs) that trigger a direct antitumor response, as well as their mobility between lymphoid and nonlymphoid tissues, and the modulation of co-stimulators such as cytokines and chemokines in controlling inflammation that is relevant to the prolonged antitumor effect [242,246,247]. In the context of the antitumoral immune response against HPV-positive tumors, a pivotal point to achieve therapeutic efficacy is the modulation of DCs and the promotion of increased cellular immunity, particularly of tumor-specific cytotoxic T lymphocytes. To this end, the studies for the treatment of precancerous lesions and cancers associated with HPV have used E6 and E7 as antigenic targets, as these oncoproteins are strongly expressed during the tumor formation process [247,248]. Additionally, combinatorial approaches with DCs and conventional methods like chemotherapy have shown promising results in reducing tumor burden [249].

Various types of DC-based vaccines have been investigated, including those using DCs pulsed with peptides derived from HPV, such as E6 and E7, as well as those using DCs transduced with DNA or viral vectors encoding heterologous antigens. For instance, clinical trials using DC-based therapeutic vaccines in patients with HPV+ and metastatic cervical cancer demonstrated the induction of immune responses and regression of cancerous lesions [179]. Pulses DC-based vaccination has also been applied in patients with stage IB or IIA cervical cancer, resulting in the expansion of HPV-specific T cells and improved clinical responses [181]. Additionally, the study by Thornburg et al. (2000) evaluated the use of pulsed DCs transfected with RNA-encoding of HPV-16 E6 and E7, showing that the stimulation of specific T cells was effective in lysing cervical cancer cells [250]. In another study, Bolhassani et al. (2019) used DCs, and mesenchymal stem cells (MSC) modified with sHsp27 and sHsp20, associated with the E7 antigen of HPV-16 in a murine model to develop therapeutic vaccines. The data revealed that DCs pulsed with the constructs could stimulate high IgG2a, IgG2b, IFN-γ, and IL-10 levels with a Th1 profile-oriented response [251]. Moreover, camelid-derived single-domain antibody fragments have shown promise as targeted delivery vehicles for antigens bound to them [252]. One study targeted VHH+ CD11b-E7 to murine DC2.4 cells, and the mice immunization resulted in more tumor-infiltrating CD8+ lymphocytes in HPV tumor-bearing mice [252]. 

In addition to the isolated application, the regimen with DCs in combination with conventional methods (chemotherapy or radiotherapy) was used to treat lesions associated with HPV. Dhandapani et al. (2021) used pulsed DCs derived from human monocytes combined with the rhSPAG9 antigen and investigated the combinatorial effect of cisplatin chemotherapy. This combination provoked a potent Th1 response and stimulated the proliferation of cytotoxic T lymphocytes, helping to reduce the tumor burden [249]. As a result, these findings shed light on the enormous potential of DC-based vaccines as personalized and effective treatments, representing a promising avenue for advancing cancer immunotherapy and personalized treatments for HPV-related cancers.

## 9. Conclusions and Perspectives

In this way, modulating antigen-presenting cells, particularly macrophages and dendritic cells, has excellent potential for immunotherapeutic intervention in HPV-related cancers. Targeting TAMs and mMDSCs and harnessing the antigen-presenting capabilities of DCs may enhance antitumor immune responses and improve clinical outcomes. However, more research is needed to optimize these strategies, including developing specific delivery systems and identifying suitable markers to characterize immune cells for clinical trials to evaluate the safety and efficacy of these immunomodulatory interventions in HPV-positive cancer patients. Future studies should focus on refining the various immunomodulatory strategies described, finding new targets, and refining combinations of techniques. Advances in nanotechnology and targeted delivery systems may facilitate specific immune cell modulation in the TME. Furthermore, a better understanding of the interactions between immune cell subsets and their interactions with HPV-infected cells will provide valuable insights for developing personalized vaccines. In addition, the combination with other therapies, such as immunosuppressive agents and checkpoint inhibitors, may enhance the therapy in HPV-related cancers have further increased.

## Figures and Tables

**Table 1:** Current state of antibody-based strategies for immunotherapy in HPV-related cancers: targeting receptors and neoantigens.

Target	Antibody-Based Strategy	Mechanism of Action	Clinical Status	References
EGFR	Cetuximab	Monoclonal antibody targeting EGFR	FDA-approved for HNSCC	[158,159,160]
	Panitumumab		FDA-approved for colorectal cancer	[161,162]
NKG2D	Monalizumab	Monoclonal antibody targeting NKG2D ligands	Under clinical evaluation for various cancers	[157,163,164]
PD-1	Pembrolizumab	Monoclonal antibody targeting PD-1	FDA-approved for various cancers	[165,166,167]
	Nivolumab			[64,168,169]
CTLA-4	Ipilimumab	Monoclonal antibody targeting CTLA-4	FDA-approved for various cancers	[166,170,171]
CD40	Selicrelumab	Monoclonal antibody targeting CD40	Under clinical evaluation for solid tumors	[172]
Tumor neoantigens	NEO-PV-01	Personalized vaccine targeting tumor neoantigens	Under clinical evaluation for various cancers	[173,174]
	Nivolumab + Ipilimumab	Combination therapy targeting PD-1 and CTLA-4	FDA-approved for MSI-H/dMMR solid tumors	[175,176]

**Table 2:** Dendritic cell subtypes and their impacts in HPV-positive cancers.

DC Subtypes	Features	Impact on HPV+ Cancers	References
cDC1	Express CLEC9A, CADM1, XCR1, CD141	- Involved in antitumor response mediated by CD8+ T cells	[241]
		- Correlated with better cancer prognosis	
cDC2	Express SIRPα, CD1c (BDCA1), CLEC10A (CD301a)	- Specialized in CD4+ T cell activation	[242,243]
		- Involved in the polarization of tumor-infiltrating lymphocytes (TILs) concerning Th1 and Th17 antitumor phenotypes	
MoDCs	Result from inflammatory processes	- Rapid differentiation	[244]
		- Positively regulates immune signals for priming of T cells	
pDCs	Express CD4, CD123, CD303, CD304, BDCA-2, HLA-DR, TLR7/TLR9	- High production of type I interferon (IFN-I)	[242,245]
		- Limited antigenic presentation and regulatory role	
		- Can be used as auxiliaries to favor the antitumor response	

Description: C-type lectin domain containing 9A (CLEC9A), cell adhesion molecule 1 (CADM1), X-C motif chemokine receptor 1 (XCR1), thrombomodulin (CD141); signal regulatory protein α (SIRPα), CD1c (BDCA1), CLEC10A (CD301a).

## Data Availability

Not applicable.
